# Outcomes after rotator cuff repair in the elderly as assessed by the American Shoulder and Elbow Surgeons shoulder score

**DOI:** 10.6061/clinics/2020/e1817

**Published:** 2020-08-14

**Authors:** Yushun Fang, Qingsong Zhang

**Affiliations:** Department of Orthopaedic Surgery, Wuhan Fourth Hospital, Wuhan City, China.

**Keywords:** Rotator Cuff Repair, ASES, Elderly, Meta-Analysis

## Abstract

Rotator cuff tears are common among the elderly, and studies on the outcomes after rotator cuff repair in the elderly are limited. We carried out this meta-analysis with systematic literature search, aiming to clarify the outcomes after rotator cuff repair in the elderly as assessed by the American Shoulder and Elbow Surgeons (ASES) shoulder score.

We conducted a literature search through October 2019 in PubMed and EMBASE databases and performed meta-analysis to calculate the summary mean difference comparing the post- and pre-operation ASES scores under both fixed-effect and random-effect models.

Among 4978 studies identified through literature search, four studies (two in the United States, one in France, and one in Republic of Korea) were eligible for the meta-analysis, including 282 patients who were aged over 70 years. These studies had low heterogeneity as measured by Cochran’s Q test (*p*=0.88) and I^2^ statistic (0%). The ASES scores on average increased by 39.7 (95% confidence interval 28.3-51.1, *p*<0.001) after rotator cuff repair, in both fixed-effect and random-effect models. No substantial publication bias was indicated.

Our findings suggest improved outcomes after rotator cuff repair in the elderly population as measured by the ASES score, and such improvements have been consistent in previous studies.

## INTRODUCTION

Rotator cuff tears are common conditions in all age groups, but the risk increases with age (1,2). Particularly, the severity of rotator cuff tears, such as tear size, may be higher among the elderly and is associated with increased pain and impaired quality of life in these patients (3,4). In spite of the certain risk of failure, there has been increasing desire to remain physically active with high levels of function in the elderly patients (5,6). This would justify the surgical repair of rotator cuff tears in the elderly patients.

A few previous studies have evaluated the outcomes after rotator cuff repair in the elderly patients, and the results vary between populations, and particularly are dependent on the tools used for assessing the post-operation outcomes (1,6-10). Among the used assessment tools, the American Shoulder and Elbow Surgeons (ASES) shoulder score is one of the most validated outcome reporting measure, which can be used in patients with a wide range of shoulder diagnoses including rotator cuff injuries (11). The ASES score includes both physician-rated and patient-reported components, and the final score is a summary measure of both pain and functional portions (12).

Aiming to clarify the outcomes after the repair of rotator cuff tears in the elderly, specifically as measured by the ASES score, we conducted this meta-analysis of published original studies with a systematic literature search. We also evaluated the heterogeneity and potential publication bias in previous studies.

## METHODS

### Literature Search

We performed a systematic literature search in PubMed and EMBASE until October 2019 without language restriction. A search strategy combining the following keywords was used to identify potentially eligible publications: (injury OR injuries OR repair OR tears) AND rotator cuff. All these terms were searched in TITLE/ABSTRACT. We also reviewed the reference lists of eligible original articles and previous systematic reviews to identify additional studies (1,2,6-8,10).

### Study Selection

We considered studies meeting the following criteria as eligible: (1) cohort studies published as original articles, (2) measuring outcomes before and after rotator cuff repair by the ASES score, (3) participants including patients who were aged 70 years or older, and (4) providing information necessary to estimate the mean difference comparing post- and pre-operation scores, with a measure of statistical uncertainty (e.g. confidence interval, standard error, variance, or *p*-value).

### Data Extraction

We extracted the following essential information from the included studies into a Microsoft Excel worksheet by one author and checked by a second author: (1) study design and characteristics (first author, year of publication, study setting, follow-up period, and number of participants), (2) distribution of sex and age at entry, (3) type of operation (arthroscopic or open, single or double row), (4) patients’ characteristics, and (5) main results.

### Assessment of Study Quality

We assessed the quality of included studies with the help of the Newcastle-Ottawa Scale (NOS) for assessing the quality of non-randomized studies in meta-analyses. This scale assesses the study quality in terms of selection of participants, comparability, and outcome ascertainment. A score ranging from zero to nine is awarded to each study with higher scores indicating higher study quality (13).

### Meta-Analysis

We conducted the meta-analysis for mean difference comparing the post- and pre-operation ASES scores under both fixed-effect and random-effect models. We examined the between-study heterogeneity by the Cochran’s Q test and I^2^ statistic. We considered a *p*-value <0.10, instead of <0.05, in the Q test as statistically significant, and this threshold is widely used in meta-analyses (14). An I^2^ larger than 50% was judged as considerable heterogeneity across studies (14,15). We checked the potential publication bias by visual inspection of funnel plots and the Begg’s and Egger’s tests (16,17). We used the software Comprehensive Meta-Analysis version 3 (Biostat, Inc. Englewood, New Jersey) for all statistical analyses.

## RESULTS

### Literature Search and Study Characteristics

**Figure 1:**
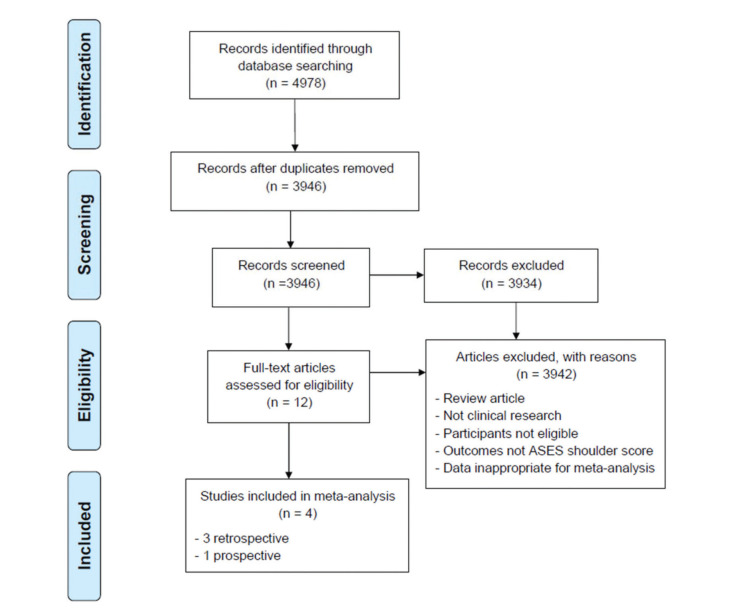
Flow chart of selection of eligible studies.

We identified 4978 studies through the initial search in the databases, among which four studies fulfilled the inclusion criteria and were included in this meta-analysis (6-8,10). A flowchart of selection of eligible studies is displayed in Figure 1. Three of the eligible studies were retrospective studies (6,7,10), while the remaining one was a prospective study (8). These studies included two conducted in the United States, one in France, and one in the Republic of Korea. These studies included 282 patients who were aged over 70 years. Arthroscopic operation (involving both double row and single row) was used in three studies conducted in Western populations (6-8), while open surgery (double row only) was used in the remaining study in the Republic of Korea (10). All patients in the three Western studies (6,7,10) and 93.5% of patients in the Korean study (8) had full-thickness tears. Two studies reported the distribution of tear size, and the proportion of large to massive tears ranged from 18% to 40% (7,10). Only one study reported the number of tendon anchors, which ranged from 1 to 5 (2-3 in 59%, and 4-5 in 31% of patients) (8). The detailed characteristics of the eligible studies are shown in Table 1. The included studies showed good study quality with the NOS quality scores of 8-9 (Table 2).

### Meta-analysis

**Figure 2:**
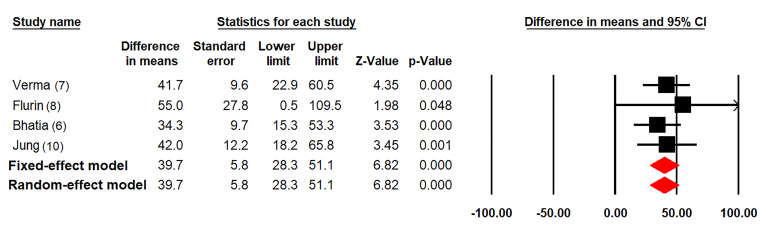
Meta-analysis of the mean difference comparing the post- and pre-operation American Shoulder and Elbow Surgeons shoulder score after rotator cuff repair. CI: confidence interval.

All of the four studies individually reported improved outcomes comparing the post- and pre-operation ASES scores. Meta-analysis of these studies generated an average increase by 39.7 (95% confidence interval 28.3-51.1, *p*<0.001) in ASES score after rotator cuff repair in both fixed-effect and random-effect models (Figure 2). Analysis restricted to the three studies in Western populations involving arthroscopic surgery revealed a similar effect size (mean difference 39.0, 95% confidence interval 26.0-52.0, *p*<0.001) under both fixed-effect and random-effect models.

### Heterogeneity and Publication Bias

**Figure 3:**
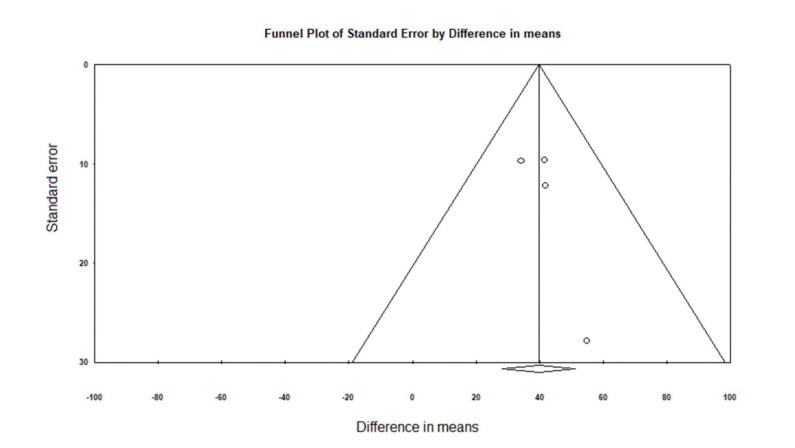
Funnel plot of the standard error by mean comparing the post- and pre-operation American Shoulder and Elbow Surgeons shoulder score.

The heterogeneity tests indicated very low heterogeneity across studies as measured by Cochran’s Q test (*p*=0.88) and I^2^ statistic (0%). No evident publication bias was indicated by visual inspection of funnel plot (Figure 3) or the Begg’s and Egger’s tests (*p*=0.263 and *p*=0.497, respectively).

## DISCUSSION

This meta-analysis with systematic literature search summarized the existing evidence regarding the outcomes after rotator cuff repair in elderly patients specifically as measured by the validated ASES score. Our results suggested improved outcomes after rotator cuff repair in the elderly population as measured by the ASES score, and such improvements were consistent in previous studies.

To the best of our knowledge, this study is the first quantitative synthesis of existing evidence regarding the outcomes after rotator cuff repair in elderly patients as measured by the validated ASES score. There are several strengths of the present study, including an extensive search strategy to identify all relevant publications, appropriate meta-analysis under both fixed-effect and random-effect models, and low heterogeneity across studies. However, there are also some limitations of this study. First, we only identified four previous studies, but the statistical power is sufficient already in the individual studies, and even improved in the meta-analysis. Second, arthroscopic operation was used in most of the studies, and thus, our findings may be generalized to patients who have undergone arthroscopic surgery rather than those who underwent open surgery. Third, due to the lack of relevant data, we measured the overall ASES score only, without measuring the individual components included in the score. In addition, we did not analyze the outcomes by size of the rotator cuff tear, which warrants investigations in future studies.

Although rotator cuff tears are common in the elderly population, both the surgeons and patients have been hesitant towards surgical repair because of the various risk factors for failure, including decreased bone density, and tendon vascularity, as well as comorbidities among elderly patients (1,2). However, there have been increasing demands for surgical repair of rotator cuff injuries among these patients (2,6). This study suggests substantial improvements in the ASES score after rotator cuff repair in these patients, which would, at least to some extent, justify rotator cuff repair even in the elderly patients. However, the decision of surgical treatment should be balanced with other clinical factors, including the injury severity, existence of comorbidities, and difficulty of the surgical procedures. In addition, arthroscopic surgery may be more cost-effective than open surgery for rotator cuff repair in elderly patients (18).

A number of scales have been used to assess outcomes in patients who undergo rotator cuff repair, including the ASES score, Constant-Murley and modified Constant, Shoulder Pain and Disability Index, Single Assessment Numerical Evaluation, the Shoulder Rating Scale of the University of California, Los Angeles (UCLA), Simpler Shoulder Test, and others (1,19-21). Among these, the ASES score has been well validated in terms of reliability and responsiveness, and showed high correlation with other shoulder rating scales, including Shoulder Pain and Disability Index, the UCLA scale and Constant-Murley (19,20,22). However, the scoring methodology of ASES score is complicated and its use in clinical practice requires specialized training in the medical and research staff. We did not assess the outcomes as measured in other scales, which is out of the scope of this study. However, future meta-analyses focused on the other shoulder rating scales would be valuable as well.

## CONCLUSIONS

In summary, this systematic review and meta-analysis suggests improved outcomes after rotator cuff repair in the elderly population as measured by the ASES score, and such improvements have been consistent in previous studies. The available studies mainly involved arthroscopic surgery, and more studies are needed to assess the outcomes after open surgery for rotator cuff tears in the elderly.

## AUTHOR CONTRIBUTIONS

Fang Y and Zhang Q jointly conceived and designed the study. Fang collected and analyzed the data and drafted the manuscript. Zhang Q contributed to the interpretation of the results and critically review of the manuscript.

## Figures and Tables

**Table 1:** Characteristics of studies assessing outcomes after rotator cuff repair using ASES shoulder score.

Study	Country	Study period	Number of patients	Mean (range) of follow-up, months	Mean age at entry, years	Sex, males%	Operation type	Tear size
Verma (7)	United States	2003-2007	39	36 (24-59)	75.3	46%	Arthroscopic (DR and SR)	Small to medium 82%; large to massive 18%
Flurin (8)	France	2010-2011	135	∼12	73.9	41%	Arthroscopic (DR and SR)	NA
Bhatia (6)	United States	2005-2012	44	43 (24-95)	73	75%	Arthroscopic (DR and SR)	NA
Jung (10)	Republic of Korea	2005-2013	64	30 (24-60)	78.1	33%	Open (DR)	Small to medium 60%; large to massive 40%

ASES: American Shoulder and Elbow Surgeons; DR: double row; NA: not available; SR: single row.

**Table 2:** Newcastle-Ottawa quality scores of included studies.

Study	Selection	Comparability	Outcome	Total scores
Verma (7)	3	2	3	8
Flurin (8)	4	2	3	9
Bhatia (6)	3	2	3	8
Jung (10)	4	2	2	8
